# Visualization of Anion Vacancy Defect Annihilation in CZTSe Solar Cells by Hydrogen-Assisted Selenization with In Operando X-ray Nanoprobe Studies

**DOI:** 10.1021/acsami.4c11127

**Published:** 2024-11-15

**Authors:** Chih-Yang Huang, Shao-Chin Tseng, Wei-Chao Chen, Gung-Chian Yin, Bo-Yi Chen, Kuei-Hsien Chen, Li-Chyong Chen, Cheng-Ying Chen

**Affiliations:** a International Graduate Program of Molecular Science and Technology, National Taiwan University (NTU-MST), Taipei 10617, Taiwan; b Molecular Science and Technology Program, Taiwan International Graduate Program (TIGP), Academia Sinica, Taipei 11529, Taiwan; c Center for Condensed Matter Sciences, National Taiwan University, Taipei 10617, Taiwan; d National Synchrotron Radiation Research Center (NSRRC), Hsinchu 30092, Taiwan; e Institute of Atomic and Molecular Sciences, Academia Sinica, Taipei 10617, Taiwan; f Department of Physics, National Taiwan University, Taipei 10617, Taiwan; g Center of Atomic Initiative for New Materials (AI-MAT), Taipei 10617, Taiwan; h Department of Optoelectronics and Materials Technology, National Taiwan Ocean University, Keelung 202301, Taiwan

**Keywords:** CZTSe, anion vacancy, nano-XRF, nano-XBIC, Solar Cells

## Abstract

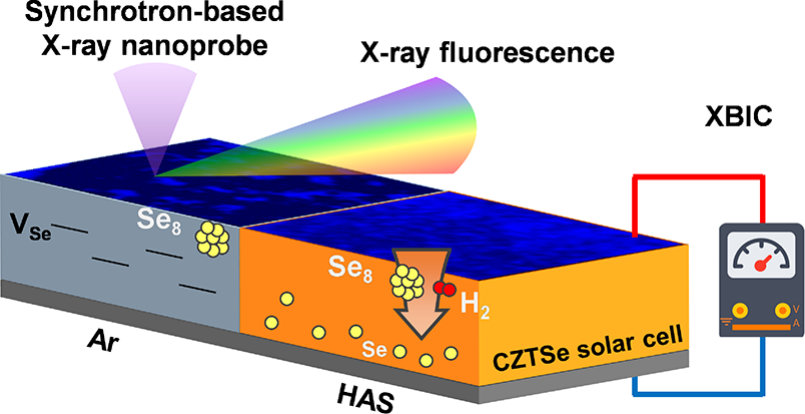

The traditional sulfur selenization process in Cu_2_ZnSn(S,Se)_4_ (CZTSSe) solar cell fabrication often results in the creation of localized anion vacancies (*V*_S_ and *V*_Se_). These vacancies are considered harmful defects as they can trap carriers generated by light, leading to reduced solar cell efficiency. Moreover, concrete evidence has been lacking on the extent of the impact caused by these anion vacancies. Our research introduces a novel approach: the hydrogen-assisted selenization (HAS) process, specifically designed to minimize localized anion vacancies in Cu_2_ZnSnSe_4_ (CZTSe) solar cells. Our investigation, utilizing current–voltage (*I*–*V*) and admittance spectroscopy measurements, provides clear insights. We observed notable improvements in carrier collection efficiency and a discernible reduction in defect states. Furthermore, there was a significant decrease in the activation energy required within the solar cell device, dropping from 184 to 145 meV. To delve deeper into the structural and compositional aspects, we employed synchrotron-based X-ray nanoprobes. Through nanoscale X-ray fluorescence and hard X-ray beam-induced current measurements, we can directly observe and document the relationship between the local compositional distribution and photocurrent activity in operando. These comprehensive results furnish strong evidence that mitigating anion vacancies in the CZTSe layer can substantially improve the power conversion efficiency of the CZTSe solar cells. This advancement not only sheds light on the critical role of anion vacancies in solar cell performance but also underscores the effectiveness of the HAS process in enhancing overall device efficiency.

## Introduction

Silicon-based solar cells, favored for their high efficiency, reliable performance, and widespread availability of raw materials, currently lead the commercial market. Yet, the production of these high-quality cells involves high-temperature processes, which not only escalates costs but also contributes to environmental pollution. Moreover, their significant thickness, approximately 100–180 μm, is necessary for absorbing sunlight effectively to produce sufficient charge carriers.^1^ This thickness renders them less ideal for integration into building photovoltaics. This limitation has prompted the development of next-generation solar cells: thin-film solar cells. Thin-film solar cells commonly use materials such as CdTe, Cu(In,Ga)(S,Se)_2_ (CIGSSe), and GaAs to attain efficiencies exceeding 20%.^2−5^ Their wide spectral response and high absorption coefficient enable them to be highly effective even in dim light conditions, offering longer effective electricity generation times compared with silicon crystal cells. However, the use of toxic, costly, and rare elements in these cells poses significant drawbacks, limiting their broader application. To address this issue, the development of new, environmentally friendly, and abundantly available solar cell materials has become a priority, with Cu_2_ZnSn(S,Se)_4_ (CZTSSe) emerging as a promising candidate. This material is seen as a potential replacement for existing thin-film photovoltaic materials owing to its exceptional optical and electrical properties, such as a high absorption coefficient (around 10^4^ cm^–1^) and a flexible bandgap between 1 and 1.5 eV.^6,7^ Despite its potential, the highest efficiency of laboratory-scale CZTSSe thin-film solar cells has only reached 14.9%,^8^ falling short of the efficiencies achieved by other thin-film cells such as CIGS (approximately 22%) and CdTe (approximately 21%).^9−11^ There are still many challenges ahead.^12^

One key challenge in CZTSSe solar cells is managing lattice defect formation in the CZTSSe absorber layer, as defects are pivotal for carrier generation, transport, and recombination.^13^ Research by Chen et al. has extensively explored the energy levels of various defects, revealing their profound influence on the electrical properties of CZTSSe.^14^ Cu vacancies and Cu_Zn_ antisite defects, for instance, act as shallow acceptor defects, enhancing p-type conductivity. By contrast, Sn_Cu_, Sn_Zn_ antisite defects, and anion vacancies (*V*_S_ and *V*_Se_) serve as deep donor defects, potentially acting as centers for carrier recombination.^15,16^ In particular, *V*_S_ and *V*_Se_ defects are known to degrade solar cell performance and are often caused by inadequate sulfo-selenization or ammonia solution etching during buffer layer growth.^17^ Multiple theoretical and experimental studies have identified these anion vacancies as performance limiters in CZTSSe solar cells.^17,18^ Zhang et al. implemented a postsulfurization treatment on the Cu_2_ZnSnSe_4_ (CZTSe) layer, aiming to passivate surface dangling bonds and anion vacancies, significantly boosting open-circuit voltage and short-circuit current.^17^ Simulations further confirm the detrimental effect of *V*_S_ and *V*_Se_ on CZTSSe solar cell performance,^18^ although direct evidence linking these specific defects to cell performance is still lacking. In conventional selenization methods, solid selenium pellets are often used due to their low melting point and minimal toxicity.^19^ However, this form of selenium is not ideal for selenization, primarily because its large molecular size (Se_8_) restricts interdiffusion in the CZTSe precursor layer.^20^ This leads to incomplete selenization and selenium-deficient areas within the CZTSe layer, adversely impacting the material’s photovoltaic behavior. Therefore, it is vital to develop methods to eliminate localized *V*_S_ and *V*_Se_ regions in the CZTSSe absorber layer, thereby enhancing the CZTSSe solar cell performance. Equally important is the need for an analytical tool that can directly probe *V*_S_ and *V*_Se_ defects and assess electrical current activity, providing crucial insights into these defects and their impact on device performance.

In this study, we aim to understand the relationship between selenium vacancies (*V*_Se_) and solar cell performance. Our approach involved the development of a hydrogen-assisted selenization (HAS) method to address local *V*_Se_ deficiencies in the CZTSe layer. Additionally, we employed nanoscale X-ray fluorescence spectroscopy (nano-XRF) combined with hard-shell X-ray-beam-induced current (nano-XBIC) measurements. This combination allowed us to monitor in real time how the elemental distribution correlates with short-circuit current (*J*_SC_) within the solar cell device. We introduced H_2_ gas during the selenization process (i.e., HAS treatment), which reacts with Se_8_ molecules, converting them into smaller H_2_Se molecules (8H_2_ + Se_8_ → 8H_2_Se). This transformation enhances selenium (Se) interdiffusion and addresses issues of local selenium deficiency. The outcome of this process showed a substantial improvement in device performance, with an increase of 13.94% in efficiency compared to devices without HAS treatment. The highest conversion efficiency achieved was 10.34% (effective area ∼0.095 cm^2^) for a CZTSe cell treated with 2.5% HAS. This treatment raised the *J*_SC_ from 33.6 to 36 mA/cm^2^, indicating that selenium vacancies likely serve as recombination centers, contributing to carrier losses. To further explore this, we utilized a synchrotron-based X-ray nanoprobe for conducting nano-XRF and nano-XBIC. This approach helped us establish a direct correlation between the local compositional distribution and electrical current activity under *in situ* conditions. Our method provides valuable insights into the intricate relationship between defects and the performance of kesterite-based photovoltaic devices.

## Experimental Section

### Device Fabrication

The 600 nm multimetallic CZT(Cu/Zn/Sn) precursors with a nine-layer structure were deposited onto 1 μm Mo-coated soda lime glass using radio frequency (RF)-magnetron sputtering deposition. According to the recipe in our previous studies, the Cu/(Sn + Zn) and Zn/Sn composition ratios of the metallic precursor are prepared at approximately 0.6 and 0.9, respectively.^21,22^ Before selenization, the CZT precursor and 90 mg of metal Se pellets (99.99% purity, Sigma-Aldrich) were placed in a semisealed graphite box (1.5 cm × 10 cm) and put inside a quartz tube equipped with a moveable furnace system. The residual water and oxygen in the tube were evacuated by a mechanical pump (1 × 10^–4^ Torr). Then, argon gas (99.99%) with 200 sccm was pumped into the quartz tube until atmospheric pressure was reached and the furnace was preheated to 550 °C. Once the pressure and temperature were stabilized, the furnace was moved to anneal the graphite box for 7 min to facilitate the selenization process. Subsequently, the sample was rapidly cooled to room temperature to prevent unfavorable decomposition. The schematic of the hydrogen-assisted selenization system is shown in Figure S1. For HAS-2.5% and HAS-5% samples, an additional 5 and 10 sccm of hydrogen (99.99%) were pumped into the tube during the annealing process. After the hydrogen-assisted selenization process, the ratios of Cu/(Sn + Zn), Zn/Sn, and Se/(Cu + Zn + Sn) change to 0.95, 1.32, and 0.9, respectively. It is hard to distinguish the difference in selenium (Se) composition among all kinds of films with different treatments by energy-dispersive X-ray spectroscopy. The detailed element compositions of the precursor and CZTSe thin film are shown in Table S1. After the selenization process, the samples were rinsed in potassium cyanide solution (KCN) (10 wt %) to remove the harmful secondary phase (Cu_2_S(e)). Subsequently, a 45 nm-thick cadmium sulfide (CdS) layer was deposited as a buffer layer using the chemical bath deposition method, and the samples were kept at 200 °C on a hot plate for a soft-baking treatment.^23^ Next, 30 nm-thick ZnO (99.99%) and 300 nm-thick ITO (99.99%) layers were deposited by RF-sputtering deposition. A 300 nm-thick Ag top electrode and a 100 nm-thick MgF_2_ were deposited using thermal evaporation. The device with a total area of 0.105 cm^2^ was defined by shadow masks and mechanical scribing (cell effective area: ∼0.095 cm^2^).

### Characterization

The structures of the CZTSe films were examined by X-ray diffraction (XRD, Bruker) and Raman scattering spectroscopy (HORIBA, JOBIN YVON Lab RAM H800). The morphologies and selenium distributions of the CZTSe thin films were investigated by field emission scanning electron microscopy (SEM, JEOL JSM-6500) equipped with energy-dispersive X-ray spectroscopy (EDS, Oxford Instruments). The compositions of the CZTSSe thin films were characterized by X-ray fluorescence (XRF, XRF-1800, Shimadzu Scientific Instruments). The devices’ entire structures and interfacial element distributions were analyzed by transmission electron microscopy (TEM, JEOL JEM-2100, equipped with scanning transmission electron microscopy, STEM) with an operating voltage of 300 kV. The surface compositions of the CZTSe films were investigated by X-ray photoelectron spectroscopy (XPS, Thermo Scientific, Theta Probe) with monochromatic Al Kα (1486.6 eV) as the radiation source. The pass energy and energy resolution are set up as 20 and 0.1 eV, respectively. Adventitious carbon, with a binding energy calibrated to 284.8 eV, was used as the charge reference. The survey spectra of the CZTSe films with Ar, HAS-2.5%, and HAS-5% treatments are shown in Figure S2. Current–voltage measurements were conducted by a Keithley 2400 source meter with an AM 1.5G solar simulator (calibrated by a standard Si solar cell). Admittance spectroscopy (AS) was performed to measure the variation of defect state levels. Nanoscale X-ray fluorescence (nano-XRF) and nanoscale X-ray beam-induced current (nano-XBIC) measurement systems were utilized at the X-ray nanoprobe beamline TPS 23A in the National Synchrotron Radiation Research Center (NSRRC), Hsinchu Science Park, Taiwan.^24^ This combined system can concurrently process nano-XRF and nano-XBIC to assess the elemental composition and distribution as well as investigate electrical properties at the nanoscale. These measurements were conducted in an ultrahigh vacuum (UHV) environment with a pressure of 1 × 10^–8^ Torr, which is crucial for minimizing interference from gas molecules and contaminants, ensuring accurate, precise measurements. The TPS 23A beamline offered an energy range of 4.5–15 keV. The spot diameter, which refers to the size of the focused X-ray beam, was approximately 100 nm, which enables highly localized analysis and mapping of samples. This system boasts a unique design for nano-XBIC measurements, featuring a 13-channel electrical measurement system capable of evaluating up to five devices simultaneously on a single sample holder. Additionally, the system includes a thermocouple on the sample seat to monitor the temperature, which is crucial for avoiding thermal effects resulting from radiation exposure. During our experiments, the samples remained unaffected by thermal effects, thanks to the carefully calibrated light source intensity and scanning duration, ensuring that they were maintained at room temperature. The photoelectric properties of the samples were concurrently analyzed using an excited optical luminescence (XEOL) instrument equipped with an optical collection system and a spectrometer covering the UV to IR range. The beamline also incorporates an electron gun, enabling it to function like SEM for real-time location and conduct electron-beam-induced conductivity (EBIC) analysis of nanostructures for surface science. Employing a fast-scanning “fly scan” technique, this system can swiftly acquire nano-XBIC and nano-XRF images with a 100 nm beam size over a scanning area of 100 μm × 100 μm in just 4 min. This enables rapid, high-resolution mapping of the elemental and electrical properties.

## Results and Discussion

**Figure 1:**
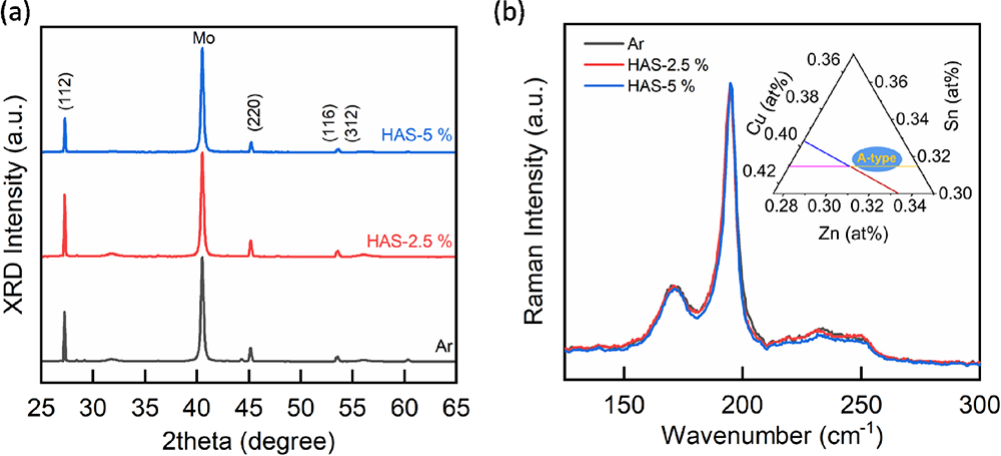
(a) X-ray diffraction pattern and (b) Raman spectra of the CZTSe thin films with a hydrogen-assisted selenization process at different H_2_ concentrations.

To evaluate the influence of the HAS method on the development of CZTSe thin films, we first performed XRD analysis, aimed at examining the crystal structure and crystallinity of the materials. For a fair comparison, all spectra were normalized and aligned with the primary peak of the cubic-molybdenum substrate at 40.5°. Figure 1a clearly shows that in all spectra, the peaks at 28.2, 45, and 53° correspond to the (112), (220), and (116/312) planes of the kesterite CZTSe structure, respectively, as indicated in the standard PDF 52-0868. The most prominent peak at 28.18° indicates a preferential growth of the CZTSe thin film along the (112) plane. Importantly, there were no significant shifts in peak positions, suggesting that HAS treatment does not affect the lattice structure of CZTSe. The full width at half-maximum (FWHM) of the (112) peaks was also calculated to assess the average grain size. The FWHM values for all samples were found to be similar at 0.136, 0.141, and 0.139°, indicating that HAS treatment does not modify the crystal size of the CZTSe thin films. There are no significant features indicating the common secondary phases, such as CuSe and SnSe_2_, corresponding to the XRD peaks at 44.2 and 60.3°, respectively. In addition, the XRD peaks at 31.8 and 56.1° indicate that the MoSe_2_ formation from the Mo back contact is not noticeable.^25^ It is important to note that this XRD analysis is limited to detecting material variations on a long-range order scale. Therefore, it may not provide a complete understanding of the effects of HAS treatment, particularly in terms of defect formation within the CZTSe thin films.

Raman spectroscopy has emerged as a more insightful technique for investigating the defect formation in CZTSe thin-film layers. Literature indicates that the vibrations within the CZTSSe crystal structure can be altered by defects such as V_Cu_, Zn_Sn_, or Zn_Cu_, leading to noticeable changes in its Raman spectra.^26^ In our study, we utilized a 633 nm excitation laser that penetrates about 100 nm into the CZTSe layer to examine the surface of the thin film. For an accurate analysis, all Raman spectra were normalized to the principal peak of CZTSe before plotting. Figure 1b demonstrates that the Raman peaks observed at 170 (A mode), 196 (A mode), 223 (E mode), and 234 (B mode) cm^–1^ are associated with the A, E, and B vibration modes, respectively.^27^ The peak at 251 cm^–1^ corresponds to cubic-ZnSe,^27^ showing a small amount of the ZnSe phase, which is attributed that all kinds of CZTS films were prepared with Zn-rich and Cu-poor compositions (the ratio of Cu/(Sn + Zn), Zn/Sn, and Se/metal changes to 0.95 and 1.32). Due to the similar XRD patterns of ZnSe and CZTSe, it is hard to identify the presence of ZnSe in the Figure 1a XRD pattern.^28^ The similarity in the Raman shape across all samples suggests that HAS treatment does not significantly affect the formation of defects, such as V_Cu_, Zn_Sn_, and Zn_Cu_. However, it is important to note that Raman spectroscopy is not capable of assessing the formation of *V*_Se_ defects. Consequently, we recognize the need to develop a new analytical tool specifically for investigating the impact of HAS treatment on the formation of *V*_Se_ defects within CZTSe thin films.

**Figure 2:**
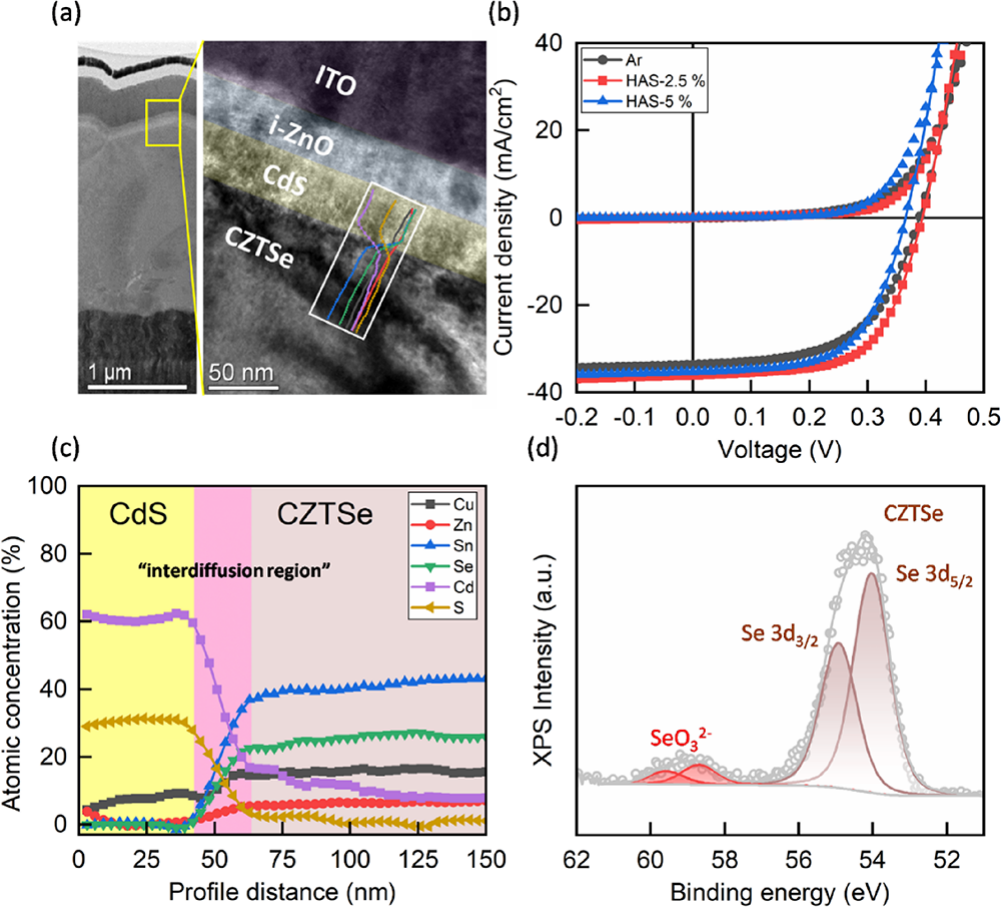
(a) Cross-sectional TEM image of CZTSe solar cells with hydrogen-assisted selenization (HAS-2.5%). (b) *J*–*V* characteristics of the CZTSe solar cells with the hydrogen-assisted selenization process at different H_2_ concentrations. (c) TEM-EDS depth profile at the CZTSe/CdS interface (HAS-2.5%). (d) XPS spectra at Se 3d for the CZTSe film with hydrogen-assisted selenization (HAS-5%).

To assess the effect of HAS on solar cell performance, we prepared three variations of CZTSe absorbers: untreated (Ar), treated with 2.5% HAS, and treated with 5% HAS. Device fabrication followed these treatments with detailed procedures described in the Experimental Section. Figure 2a presents a comprehensive STEM image of the completed CZTSe solar cell structure. This image clearly displays the layered structure: Mo/CZTSe/CdS/ZnO/ITO/Ag, confirming the successful assembly of the device. We then conducted current–voltage (*J*–*V*) characteristic measurements under dark conditions and 1 sun illumination (with an AM 1.5G filter) to evaluate the performance of the fabricated solar cell devices. For a thorough analysis, 10 devices were produced for each level of hydrogen treatment. The average results are depicted in Figure 2b, and detailed performance parameters are summarized in Table 1. The *J*–*V* curve analysis shows that the device with 2.5% HAS treatment exhibited notable improvements in the fill factor and current density compared with the Ar-treated devices. Specifically, the fill factor increased from 57.5 to 62%, and the short-circuit current density (*J*_SC_) improved from 33.67 to 36.07 mA/cm^2^. The enhancement in the fill factor can be attributed to a reduction in series resistance (*R*_S_) from 2.41 to 1.95 Ω cm^2^ and an increase in shunt resistance (*R*_Sh_) from 244.6 to 341.7 Ω cm^2^. We believe that the improved *J*_SC_ is primarily due to the reduction of localized *V*_Se_ regions in the CZTSe thin film from the HAS process. As *V*_Se_ is a known deep-level defect that can significantly increase carrier recombination and reduce photocurrent collection, mitigating these regions can effectively boost current density.^14,17^ As a result of these improvements, the average efficiency of devices treated with 2.5% HAS showed a significant improvement of 14%, achieving an average efficiency of 8.82%. This increase underscores the beneficial impact of HAS on the CZTSe solar cell performance. However, direct evidence explicitly linking the removal of localized *V*_Se_ defects to the enhancement of *J*_SC_ is still lacking.

**Table 1:** Photovoltaic Parameters of CZTSe Solar Cells with Different Concentrations of the HAS Treatment^a^

device	*V*_oc_ (V)	FF (%)	*R*_S_ (Ω·cm^2^)	*R*_SH_ (Ω·cm^2^)	*J*_sc_ (mA/cm^2^)	η (%)
Ar	0.39 ± 0.03	57.47 ± 5.89	2.41 ± 0.45	244.61 ± 121.5	33.67 ± 0.97	7.59 ± 1.3
HAS-2.5%	0.39 ± 0.01	62 ± 3.02	1.95 ± 0.14	341.71 ± 166.3	36.07 ± 0.44	8.82 ± 0.61
HAS-5%	0.37 ± 0.01	59.94 ± 1.19	1.9 ± 0.09	220.75 ± 51.9	35.34 ± 0.38	7.75 ± 0.33
best (HAS-2.5%)	0.40	63.89	1.76	391.62	36.58	9.35 (10.34) eff.

a Statistics from 10 cells for each condition. The best efficiency of HAS-2.5% is calculated by effective area (0.095 cm^2^).

It is important to note that the enhancements in the solar cell performance did not progressively increase with higher hydrogen concentrations in our study. Surprisingly, the open-circuit voltage (*V*_OC_) began to decrease when the hydrogen concentration exceeded 5%. This decrease is likely due to increased selenium (Se) interdiffusion into the CdS layer. Figure 2c showcases our STEM–EDS line profile measurements, revealing a distinct “interdiffusion region” (marked in the pink region) at the CdS–CZTSe interface, which is attributed to the soft-baking treatment after CdS deposition.^23^ Our previous study investigated the correlation between the interdiffusion region and solar cell performance by varying soft-baking temperatures.^23^ The results reveal that controlled interdifussion could enhance solar cell performance by optimizing the interface characteristics.^23^ Other forms of literature also suggest that while a certain level of phase mixing at the interface can reduce recombination, thereby improving the efficiency of chalcogenide-based photovoltaic devices,^29,30^ excessive diffusion can actually degrade the device performance. Figure S3 clearly indicates that the hydrogen-assisted selenization sample exhibits a diffusion region (22 nm) near the CdS/CZTSe interface that is wider than that of the Ar sample (15 nm). To understand the variation in the width of the interdiffusion region, we conducted an XPS analysis to measure the elemental composition of the CZTSe surface. The results reveal that applying HAS treatment with 5% hydrogen led to a notable change: the emergence of a SeO_3_^2–^ peak in the XPS spectra of these samples. Compared with all XPS spectra at Se 3d for the CZTSe films (as shown in Figure S4), the intensity of the SeO_3_^2–^ peak gradually increases with the hydrogen concentration, indicating more selenium (Se) accumulation on the surface of the CZTSe thin films. From a thermodynamic perspective, this increased selenium (Se) accumulation at the CdS/CZTSe interface would likely enhance more selenium diffusion into the CdS side. According to our previous study, it has been demonstrated that soft baking can promote the interdiffusion of cadmium (Cd) and sulfur (S), potentially forming an n-type inversion layer at the interface that can significantly reduce interfacial recombination, leading to improving device performance.^23^ However, excessive selenium (Se) diffusion can be counterproductive. It may lead to the forming of a CdSe layer with a narrow bandgap at the interface, causing unfavorable band alignment and significant carrier recombination.^31^ Therefore, controlling the hydrogen concentration during the HAS process is crucial. Excessive selenium diffusion must be avoided to maintain optimal band alignment at the interface and ensure the best performance of the photovoltaic device.

**Figure 3:**
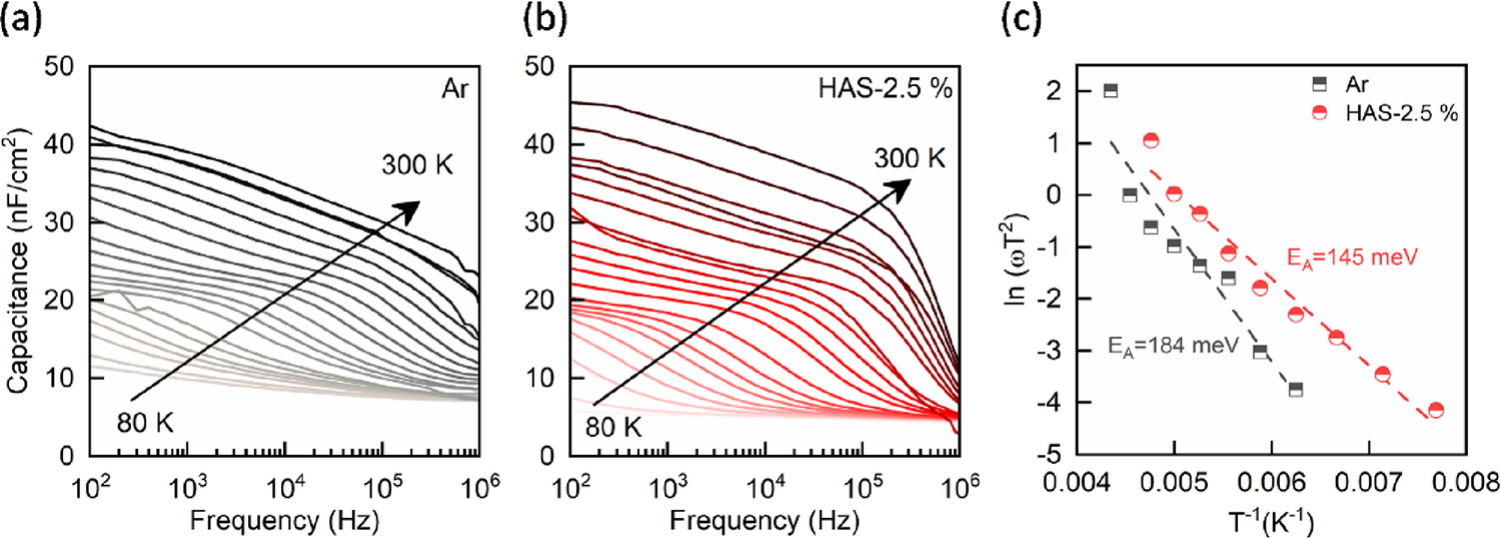
Admittance spectra of CZTSe devices (a) without and (b) with hydrogen-assisted selenization. (c) Defect level derived from the Arrhenius plot of the admittance spectra.

**Figure 4:**
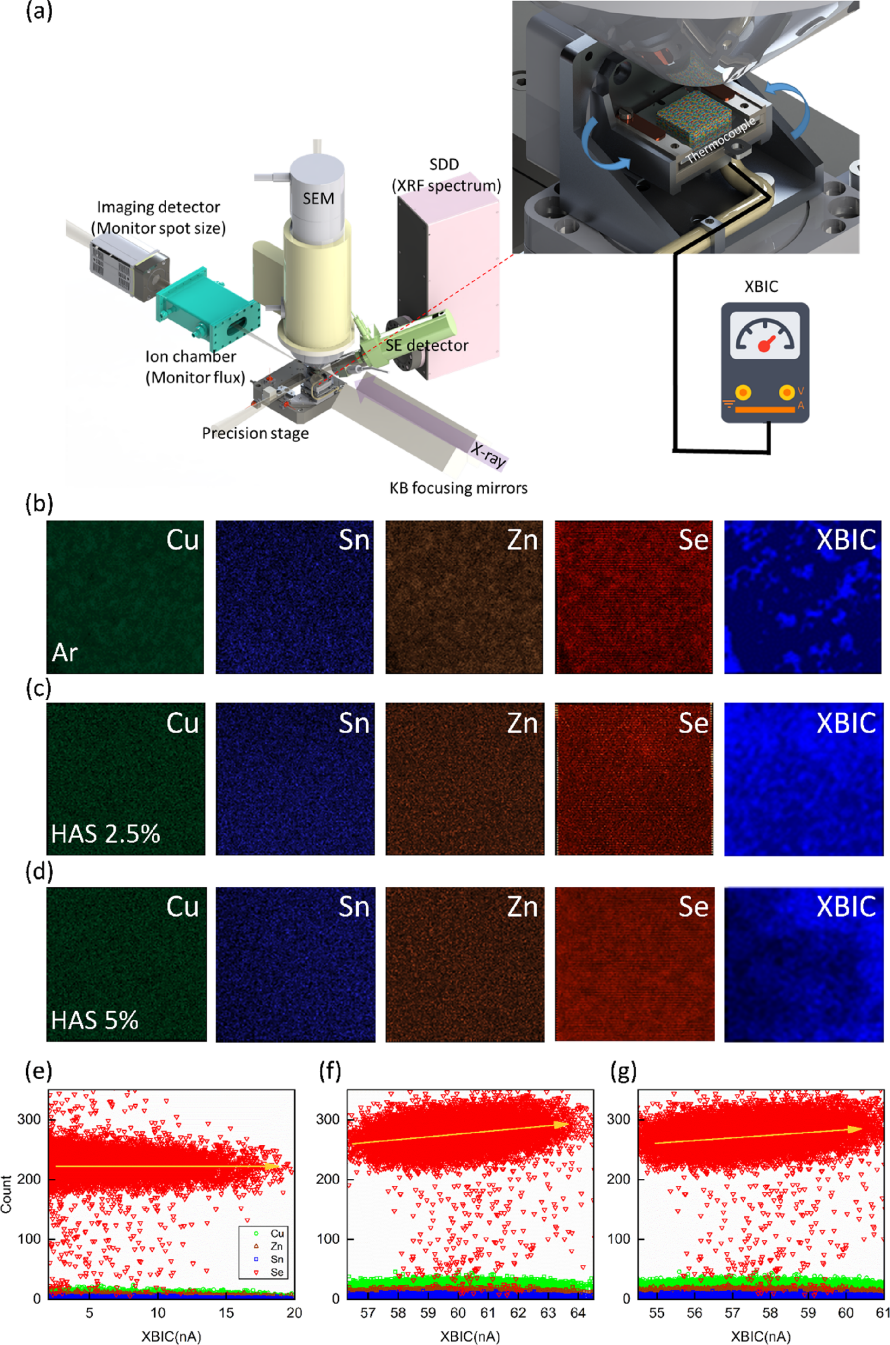
(a) Schematic of the nano-XBIC and nano-XRF measurement experimental setup. (b–d) Nano-XRF and nano-XBIC area scan mappings (100 μm × 100 μm) for samples treated with Ar, HAS-2.5%, and HAS-5%, respectively. (e–g) Correlation analysis of composition and nano-XBIC for samples treated with Ar, HAS-2.5%, and HAS-5%, respectively.

To substantiate that HAS indeed mitigates localized *V*_Se_, we conducted admittance spectroscopy (AS) measurements on both Ar- and HAS-2.5%-treated devices. AS is a widely used electrical characterization technique for investigating defect states in materials. This method involves measuring the variation in capacitance of a photovoltaic device across different frequencies (from 10^2^ to 10^6^ Hz) and temperatures (ranging from 80 to 300 K), enabling the analysis of the presence and characteristics of trap defects.^23,32−34^ According to the Kimerling model, capacitance in the high-frequency region mainly reflects the contribution from free carriers. By contrast, the low-frequency region represents a mix of free carriers and carriers trapped in deep defects. By examination of AS data, we can determine the number and energy levels of these defect states in the devices. Figure 3a and Figure 3b show the AS spectra for Ar- and HAS-2.5%-treated devices, respectively. To calculate the activation energy (*E*_a_) of the deep defects, we created an Arrhenius plot using the inflection point of each AS spectrum. These points, determined by the angular frequency at the maximum of ωd*C*/dω, were utilized for the Arrhenius plot. The plot was fitted using the following equation:^35^
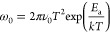
1Here, ω_0_ is the inflection point frequency; *E*_a_ represents the energy difference between the defect state and the valence band edge; ν_0_ is the pre-exponential factor, which includes temperature-independent components like the defect capture cross section for holes (σ_p_), thermal velocity (υ_th_), and the effective density of states in the valence band (*N*_V_). Analysis of the Arrhenius plots (Figure 4c) revealed a decrease in activation energy from 184 to 145 meV. This decrease, ascertained from the Arrhenius plots, can be interpreted as the average depth of the defect states, suggesting that HAS treatment effectively reduces the average depth of these states. Combined with previous characterization results, this finding strongly suggests that HAS treatment’s ability to reduce *V*_Se_ is a contributing factor to the observed improvements in photovoltaic device performance.

This study focuses on unveiling the link between selenium vacancies (*V*_Se_) and the performance of solar cells. We employed nano-XRF and nano-XBIC techniques to directly observe and analyze the relationship between the local compositional distribution and photocurrent activity within the solar cells. A key tool in this research is the synchrotron radiation X-ray nanoprobe beamline, which utilizes high-energy X-rays from a synchrotron to probe nanomaterials. Figure 4a presents a schematic of this technique, encompassing the synchrotron radiation source, X-ray optics, the sample, and the detectors. The X-ray optics are fine-tuned to the sample’s size and properties, concentrating the X-ray beam appropriately. The silicon drift detector (SDD) is utilized to collect nano-XRF spectra, providing elemental composition and distribution information. Additionally, the system integrates an electron gun, enabling it to function like an SEM for real-time location and conduct EBIC measurement. This combined approach of nano-XRF and nano-XBIC measurements offers a powerful means to understand the effects of *V*_Se_ on the performance of solar cells under real-world conditions. Further details of the measurements are outlined in the Characterization section.

Figure 4b displays the results from a large-area scan (100 μm × 100 μm) using 13.5 keV X-rays on the device treated with argon (Ar). The Ar-treated scan revealed an uneven distribution of key elements, such as Cu, Sn, Zn, and Se. Notably, these elements appear to form clusters or aggregates, indicating an inhomogeneous composition within the material. The corresponding photocurrent, as measured by nano-XBIC, is notably low in this scenario. This observation suggests that defects within the material significantly impact charge carrier transport and recombination. The ability of nano-XBIC to map the spatial distribution and density of these defects is crucial to understanding their effect on the electrical properties of the material. By contrast, the results of a similar large-area scan on the devices treated with HAS-2.5% and HAS-5% in Figure 4c,d show the uniform distributions of Cu, Sn, Zn, and Se, indicating the more homogeneous material composition. This homogeneity is attributed to the HAS treatment [8H_2_ + Se_8_ (large molecule) → 8H_2_Se (smaller molecule)], which enhances selenium (Se) interdiffusion and reacts with multimetallic CZT(Cu/Zn/Sn) precursors, addressing issues of local selenium (Se) deficiency. Correspondingly, there is a noticeable increase in the photocurrent, indicative of superior photoelectric characteristics. The uniform distribution of elements and the enhanced photocurrent in Figure 4c suggest that HAS-2.5% treatment improves charge carrier transport and recombination, leading to better device performance. The study also includes top-view nano-XRF analysis, focusing on the local composition of a specific solar cell. This analysis revealed that Se tends to segregate around certain defects within the absorber layer.^36,37^Figure 4e–g offers atomic concentration maps for elements in the CZTSe layer alongside the calculated nano-XBIC signal. Through correlation analysis, it was observed that the photoelectric current is predominantly influenced by the positive correlation between the Se elements and their associated defects. This correlation suggests that the presence and distribution of Se, especially in relation to defects, are crucial in determining the photoelectric characteristics of the solar cell. Therefore, understanding and managing Se behavior and its related defects could be key to enhancing solar cell performance. Overall, nano-XRF analysis in this study offers valuable insights into the local composition and the interplay between specific elements and defects. These findings are instrumental in elucidating the factors that influence the photoelectric properties of solar cells.

## Conclusions

In this study, we effectively addressed selenium vacancies in CZTSe thin films by adopting a hydrogen-assisted selenization (HAS) method. This technique led to a more uniform surface morphology of CZTSe thin films, effectively reducing the number of localized areas lacking selenium. This improvement in film quality translated into an enhanced performance of the CZTSe solar cells. Notably, there was a significant increase in the short-circuit current density (*J*_SC_), which increased from 33.6 to 36.1 mA/cm^2^. As a result of these changes, the overall efficiency of the solar cells, measured over the effective cell area, achieved a notable 10.36%. Admittance spectroscopy (AS) played a key role in our study, offering deep insights into how HAS treatment influences defect states within solar cells. The AS results indicated a decrease in activation energy for HAS-2.5%-treated devices, falling from 184 to 145 meV. This reduction points to a lowered average depth of defect levels, suggesting a more efficient charge carrier process. Further evidence supporting these findings came from nanoscale X-ray fluorescence (nano-XRF) and nanoscale X-ray beam-induced current (nano-XBIC) measurements. These techniques revealed a direct correlation between the photocurrent and the presence of selenium vacancy defects. This correlation underscores the significant role that these defects play in the overall performance of CZTSe solar cells, demonstrating how addressing these vacancies can lead to substantial improvements in solar cell efficiency.

## Supporting Information Available

Schematic of the hydrogen-assisted selenization system; composition analysis of metal precursors and selenized films using energy-dispersive X-ray spectroscopy; survey spectra of the CZTSe films with Ar, HAS-2.5%, and HAS-5% treatments; TEM-EDS depth profile at the CZTSe/CdS interface for Ar and HAS-2.5%; and the XPS spectra of Se 3d for the CZTSe films with Ar, HAS-2.5%, and HAS-5% (PDF)
