# Supplementary material for: Visualization of Anion Vacancy Defect Annihilation in CZTSe Solar Cells by Hydrogen-Assisted Selenization with In Operando X-ray Nanoprobe Studies
Source: ACS Appl Mater Interfaces. 2024 Nov 15;16(47):64656–63. doi: 10.1021/acsami.4c11127 (PMC11615846; doi:10.1021/acsami.4c11127)
Supplement: Supplementary file 1 — am4c11127_si_001.pdf [file am4c11127_si_001.pdf]

Supporting Information

Visualization of anion vacancy defect annihilation in CZTSe solar cells by hydrogen-assisted selenization with in-operando X-ray nanoprobe studies

*Chih-Yang Huang [a, b, c, †], Shao-Chin Tseng [d, †, *], Wei-Chao Chen [c, e, †], Gung-Chian Yin [d], Bo-Yi Chen [d], Kuei-Hsien Chen [c, e], Li-Chyong Chen [c, f, g, *] and Cheng-Ying Chen [c, h, *]*

a. International Graduate Program of Molecular Science and Technology, National Taiwan University (NTU-MST), Taipei 10617, Taiwan

b. Molecular Science and Technology Program, Taiwan International Graduate Program (TIGP), Academia Sinica, Taipei 11529, Taiwan

c. Center for Condensed Matter Sciences, National Taiwan University, Taipei 10617, Taiwan

d. National Synchrotron Radiation Research Center (NSRRC), Hsinchu 30092, Taiwan

e. Institute of Atomic and Molecular Sciences, Academia Sinica, Taipei 10617, Taiwan

f. Department of Physics, National Taiwan University, Taipei 10617, Taiwan

g. Center of Atomic Initiative for New Materials (AI-MAT), Taipei 10617, Taiwan

h. Department of Optoelectronics and Materials Technology, National Taiwan Ocean University, Keelung 202301, Taiwan

E-mail: tseng.sc@nsrrc.org.tw; chen.chengying.cyc@gmail.com; cychen0111@mail.ntou.edu.tw; chenlc@ntu.edu.tw

* Author to whom correspondence should be addressed.

† These authors contributed equally to this work.

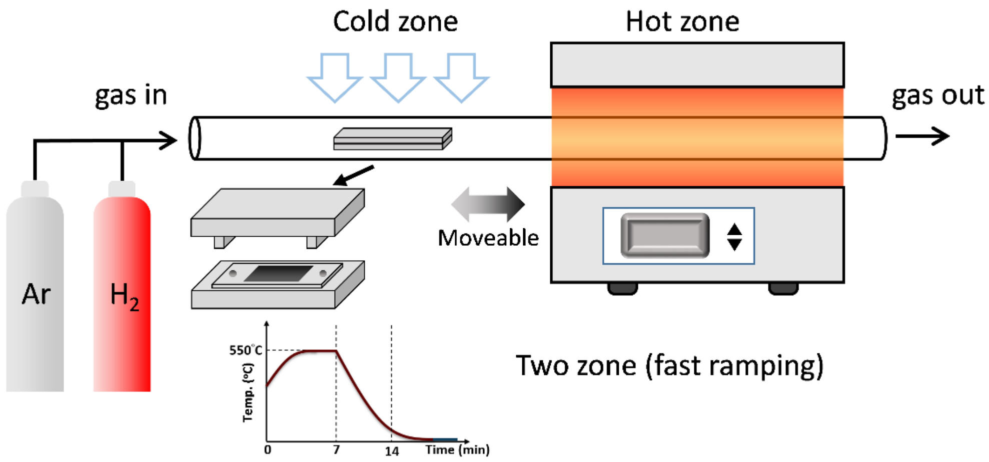

Figure S1. Schematic of the hydrogen-assisted selenization system

Table S1. Composition analysis of metal precursors and selenized films using energy-dispersive X-ray spectroscopy.

**Precursor/ CZTSe**	**Cu (at%)**	**Zn (at%)**	**Sn (at%)**	**Se (at%)**	**Cu/(Zn+ Sn)**	**Zn/ Sn**	**Se/ metal**
**Metal precursor**	36.4± 0.62	30.7± 0.35	32.9± 0.38	-----	0.57	0.93	
**CZTSe**	25.74± 0.43	15.48± 0.50	11.70± 0.37	47.94± 0.6	0.95	1.32	0.9

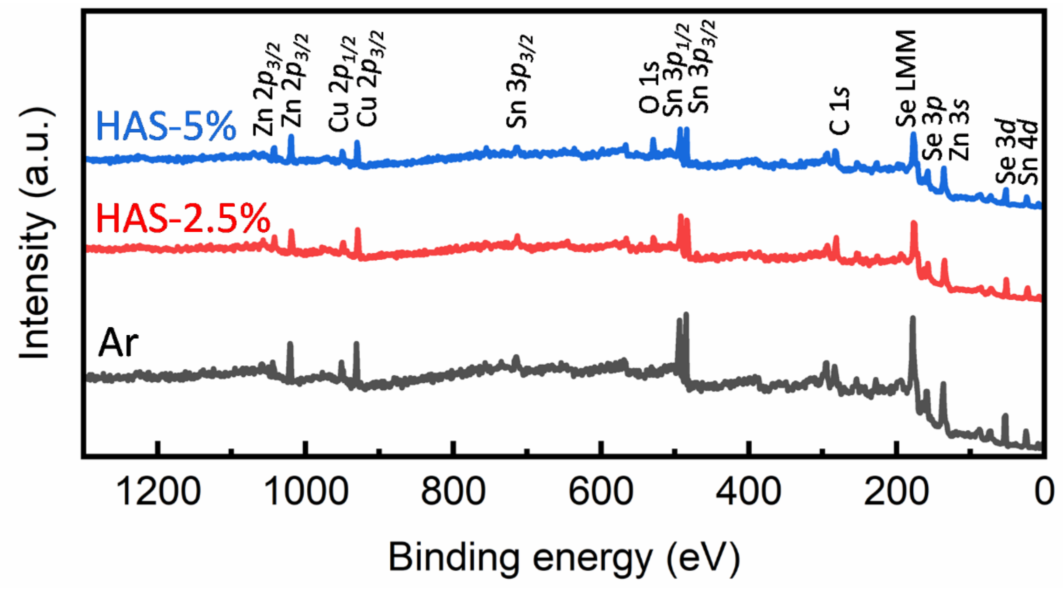

Figure S2. Survey spectra of the CZTSe films with Ar, HAS-2.5%, and HAS-5% treatments

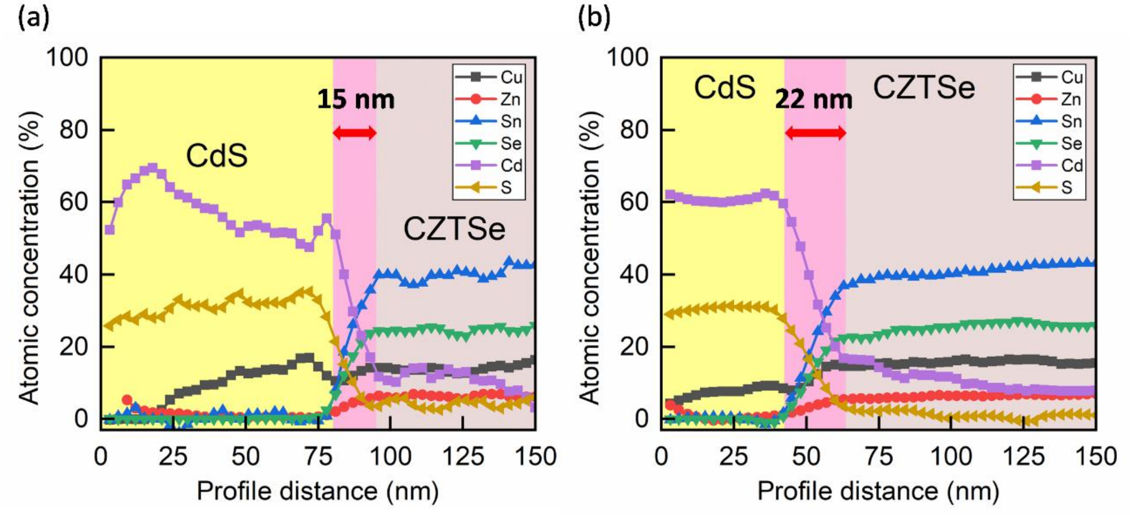

Figure S3. TEM-EDS depth profile at CZTSe/CdS interface for (a) Ar and (b) HAS-2.5%.

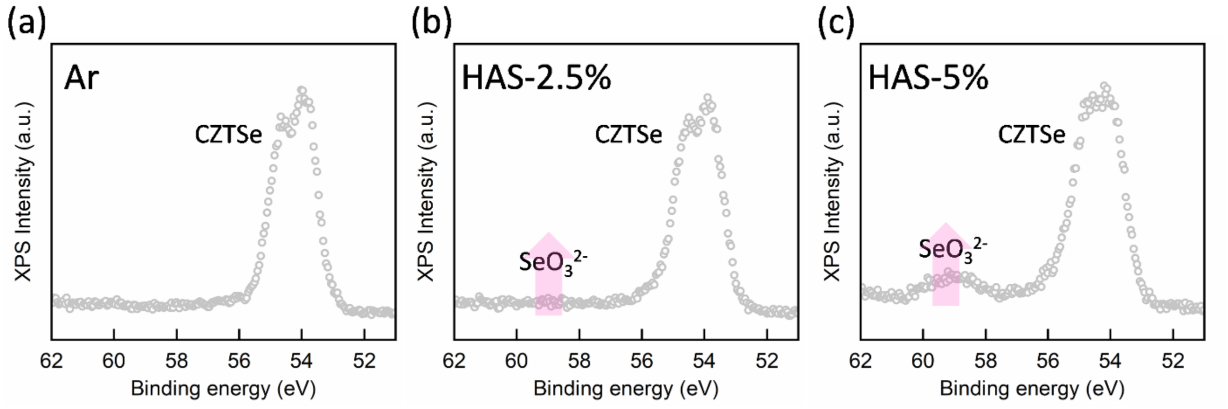

Figure S4. XPS spectra of Se 3*d* for the CZTSe films with (a) Ar (b) HAS-2.5% and (c) HAS-5%
